# Hospital to community in Wales: What is the value of optometrists playing a greater role in managing neovascular AMD and glaucoma in primary care?

**DOI:** 10.1111/opo.13397

**Published:** 2024-10-10

**Authors:** Barbara Ryan, Mari Jones, Pippa Anderson, Rhiannon Reynolds, Rebecca E. M. Nicholls, Katherine Cullen, Mark Davies, Rachel North, Bablin Molik, Carolyn Wallace

**Affiliations:** ^1^ Aneurin Bevan University Health Board Newport UK; ^2^ School of Optometry and Vision Sciences Cardiff University Cardiff UK; ^3^ Swansea Centre for Health Economics Swansea University Swansea UK; ^4^ Division of Population Medicine, PRIME Centre Wales PRIME Cardiff University School of Medicine Cardiff UK; ^5^ Faculty of Life Sciences and Education University of South Wales Pontypridd UK; ^6^ Sight Cymru Pontypool UK

**Keywords:** age‐related maculopathy, enhanced optometric service, glaucoma, hospital eye services, primary care, service pathways, value

## Abstract

**Purpose:**

To evaluate the value of enhanced optometric services for managing neovascular age‐related macular degeneration (nAMD) and glaucoma in primary care optometry services, instead of hospital eye services (HES).

**Methods:**

Seven enhanced optometric service pathways in primary care in Wales were assessed with a mixed‐methods approach: three for nAMD and four for glaucoma. The methods were a patient‐related experience measure (PREM), a Realist Review and Evaluation involving both patients and staff, a discrete event simulation model estimating the economic impact of the pathways and a workforce survey of optometrists to gauge capability and capacity.

**Results:**

Patient‐related experience measure responses (802) indicated that primary care experience was comparable to that of HES. Utilising enhanced optometric services in primary care resulted in reduced wait times compared with HES, with suspected nAMD shortened to 4–5 days and glaucoma monitoring to 5 days. Waiting lists were dramatically reduced with primary care‐based services to just three people waiting for nAMD and five for glaucoma, compared with 216 and 5691 people, respectively, in HES. Consultant ophthalmologist time was reduced from 57% to 15%–16% for nAMD services and from 48% to 22%–23% for glaucoma services. Integrating enhanced optometric services into primary care incurred a similar cost. The workforce survey confirms that optometrists possess the skills and qualifications and are willing to deliver these enhanced optometric services. The Realist Review and Evaluation revealed that clear patient communication, effective coordination and strong interprofessional communication between optometrists and ophthalmologists along with a shared electronic record are crucial to the success of this change.

**Conclusion:**

Providing enhanced optometric services in primary care for nAMD and glaucoma brings substantial benefits for the UK National Health Service and patients, including reduced waiting times, waiting lists and released HES capacity. The success of this transition hinges on clear patient communication, administrative co‐ordination and effective interprofessional communication.

**Key points**

- Integrating highly qualified primary care optometrists into the patient referral and monitoring pathways can significantly reduce wait times for neovascular age‐related macular degeneration and glaucoma, which could reduce the risk of sight loss.
- Shifting more responsibilities to primary care optometrists frees up consultant ophthalmologist time, allowing them to focus on more complex cases, thereby optimising the use of healthcare resources.
- Primary care‐based optometric services are comparable to hospital eye services in terms of patient experience and are a financially viable and scalable solution to help manage the increasing number of people with eye disease.

## INTRODUCTION

Neovascular age‐related macular degeneration (nAMD) and glaucoma are the two most common causes of sight loss in England and Wales,^1^ and they require life‐long monitoring due to their chronic sight threatening nature. Most people living with these conditions attend hospital eye services (HES), the busiest outpatient specialty in the United Kingdom,^2^, ^3^ with concerning capacity problems heightened by a projected increase of 59% nAMD and 22% in glaucoma cases over the next decade.^4^ Currently, Wales is the sole UK nation reporting detailed waiting time statistics revealing that those with these conditions often wait >25% beyond their target appointment date.^5^ The long waiting times are particularly concerning as these patients are at risk of irreversible sight loss or harm^6^ if they miss their treatment target date.

Alternative ways of delivering care are needed and have been called for by the Royal College of Ophthalmologists. Other health professionals are already involved in nAMD services in hospitals.^7^ Additionally, using virtual glaucoma clinics for low‐risk new glaucoma referrals, led by hospital optometrists, has been found to be successful.^8^ The UK National Institute for Health and Care Excellence (NICE) guidelines for glaucoma^9^ and age‐related macular degeneration (AMD)^10^ explicitly allow for delegation of greater responsibility to optometrists in referral filtering and managing low‐risk and/or stable disease.

Optometrists, like general medical practitioners and dentists, operate as National Health Service (NHS) primary care contractors and their NHS General Ophthalmic Services Contract^11^ obligates that if during sight tests, signs of injury or disease were detected, patients be referred to the HES. Some areas of Wales have enhanced services provided by optometrists in primary care for managing and/or monitoring nAMD and glaucoma. In September 2023, new legislation for the NHS General Ophthalmic Services in Wales was passed^12^ that formalises the use of enhanced optometric services in primary care in all areas and makes other changes to reconfigure eye care services to change the use of personnel, space and equipment in primary care optometry and the HES, with care delivered as close to home as possible, but with people able to access hospitals when required.^13^

UK‐based assessments have demonstrated that, with appropriate training, optometrists in primary care can manage patients effectively, and that genuine partnerships can exist between primary care optometry services and the HES.^14^, ^15^, ^16^, ^17^, ^18^, ^19^, ^20^, ^21^ However, the outcomes assessed alongside costs were not consistent between studies. The patient voice is lacking and reports of the patient experience of enhanced optometric services are scant.^14^, ^22^ Understanding the experience of patients and their opinions regarding provision of enhanced optometric services in primary care is crucial.

Optometric practices, whether independently owned or part of larger chains, exhibit varying business and clinical objectives, making it important not to presume uniform willingness to undertake additional responsibilities. Notwithstanding the possible advantages of providing primary care‐based services, it is important to gauge the readiness of optometrists to undergo training and complete higher professional qualifications^23^ and provide the extra services assuring that any service changes would be sustainable.

Against this backdrop, this research endeavours to assess the patient and NHS benefits of effective enhanced optometric services pathways for managing and/or monitoring nAMD and glaucoma provided by primary care optometry services in three areas of Wales.

## METHODS

This study used a Concurrent Nested Design (mixed model) with a predominant health economics strand and nested qualitative approach. Enhanced optometric services, with service specifications and qualifications stipulated by the health board in three health boards in Wales, were evaluated for three pathways of nAMD care:

- Traditional (nAMD‐T): If a primary care‐based optometrist suspects nAMD, the patient is referred onto HES within 1 day. All patient care is undertaken in HES.
- Referral refinement virtual review by ophthalmologists (nAMD‐VR): If a primary care‐based optometrist suspects nAMD, the patient is referred to a referral refinement centre in a primary care‐based optometry practice. Scans are uploaded onto a clinical workstation and reviewed virtually by a consultant ophthalmologist, who will either discharge the patient or refer into the hospital.
- Referral refinement optometry decision (nAMD‐OD): As nAMD VR, however, the optometrist in primary care‐based services with a Professional Certificate in Medical Retina reviews the scans and makes the decisions.

In some areas of Wales, primary care‐based optometrists in NICE compliant pathways^9^, ^10^ help monitor the lowest risk stratified glaucoma patients.^24^ This research evaluated three pathways of glaucoma care with service specifications and qualifications stipulated by the health board:

- Traditional (G‐T): Every aspect of patient monitoring is carried out in the HES.
- Monitoring with virtual review by ophthalmologists (G‐VR): Primary care‐based optometrist with a Professional Certificate in Glaucoma examines the patient and images. Results are uploaded onto clinical workstation for a consultant ophthalmologist to review virtually.
- Monitoring with virtual review and decision by hospital optometrist (G‐HOVR): A hospital optometrist with a Higher Professional Certificate in Glaucoma makes the decisions and an ophthalmologist is involved in higher risk or more complex cases.

A fourth pathway of glaucoma care emerged just after the end of data collection (named ‘G‐OD’), a primary care‐based optometrist with a Higher Professional Certificate in Glaucoma reviews the scans and results in the practice and makes the decisions with an ophthalmologist involved in higher risk or complex cases.

### Stakeholder engagement

Two of the co‐authors were ‘stakeholders’ who were involved in all aspects of the research; one chaired the Stakeholder Advisory Group. The Stakeholder Advisory Group included members that were patients, optometrists, ophthalmologists, hospital managers and voluntary sector workers, and they met four times through the project lifetime.

### Patient‐reported experience measure (PREM) and patient costs

An ‘Eye‐Care PREM’ (Supplementary S2) was given to patients at 11 primary care‐based optometry practices who provided the enhanced optometric services and three hospitals. Although the numbers of practices involved in enhanced optometric services during the period of the study varied, this was at least half of all practices providing enhanced optometric services for glaucoma and nAMD in Wales during this period. The ‘Eye‐Care PREM’ comprised of four sections: ‘(1) About you’, ‘(2) Your appointment today’, ‘(3) Your experience of the appointment’ and ‘(4) Your experiences of the appointment—COVID‐19 safety measures’. Section 1 had four demographic questions, Section 2 had 12 questions about the patient's experience before the appointment, costs for travelling to and from the healthcare facility they visited and other variables to help calculate costs such as amount of annual leave taken to attend a clinic and whether there were any carers present. Section 3 had 14 Likert scale patient experience questions and Section 4 had four questions linked to COVID safety measures. There was also space for a free text answer.

Data collection was a rolling process, starting with the primary care‐based services in Aneurin Bevan University Health Board (ABUHB) on 15 May 2021 and ending on 11 January 2023 in a hospital in Cwm Taf Morgannwg University Health Board (CTUHB). The method of collection was co‐produced with the Stakeholder Advisory Group. Patients attending the services were invited to participate by staff who offered patients a questionnaire and a pen and asked them to post‐completed questionnaires into a locked box. Prepaid envelopes were available if patients wished to take the questionnaire home and posters containing information about an online version were also displayed at all sites. The research team provided initial training to staff in the participating practices and hospitals, met with service leaders and visited the sites at least twice during the period of collection to encourage them to give a PREM to every patient or encourage them to fill in the online version.

Data were input into Microsoft Excel (microsoft.com) and analysed using STATA (StataCorp LLC, version 17, stata.com). Missing data were investigated to determine if it was missing at random. Non‐parametric inferential tests were conducted to compare patient experience in the hospital service to that in primary care‐based services for nAMD and glaucoma.

### Realist Review and Evaluation

To understand patients' and professionals' beliefs and experiences about enhanced optometric services and identify the best ways to effect change of location of care, the following questions were asked:

- What matters to those with nAMD and glaucoma about the manner and place of care and why?
- How will changing services affect patients, optometrists, ophthalmologists and other stakeholders?
- To what extent do primary care‐based services matter to stakeholders in terms of their experience and preferences?

These questions were interrogated and addressed through three cycles of realist enquiry. Execution and reporting of findings was consistent with the RAMESES Reporting Standard for both Realist Synthesis and Evaluation.^25^, ^26^ Ultimately, the goal of this work package was to develop a ‘Refined Programme Theory’ of primary care‐based treatment for those with nAMD and glaucoma. This abstracted middle range theory is a theory that has been developed to provide a focused, testable and practical framework, to articulate both why and how these services work, and is a theory of both causation and implementation.^25^

During *Cycle 1*, we drew upon early health economics modelling, initial scoping of the literature and input from the Stakeholder Advisory Group to induct an Initial Programme Theory. At this stage, the IPT may consist of a simple process map or logical model that will be further elaborated upon as the Realist Enquiry progresses.^27^

*Cycle 2* further dimensionalised the embryonic Initial Programme Theory—iteratively confirming, refuting and refining the elements through a systematic review of the literature.^25^, ^28^ Remaining congruent with Realist methodology, grey literature was also included spanning 2014–2022, as well as other sources identified by the Stakeholder Advisory Group. Within Cycle 2, data were initially ‘bucket coded’ into congruent categories. These bucket codes were then examined carefully to elucidate the underpinning causative mechanisms, generating the *Context‐Mechanism‐Outcome configurations* that form the substrate of all Realist Programme Theory.^29^ The complete review protocol is available at PROSPERO (CRD42021260517).

Within *Cycle 3*, we moved from *Realist Review* into *Realist Evaluation*. The themes from the patient survey and ‘Interim’ Programme Theory that surfaced within Cycle 2 were further tested with a series of stakeholder groups in order to develop a Refined Programme Theory of primary care‐based treatment for those with nAMD and glaucoma.

The Interim Programme Theory was tested within a Realist Interviewing process^30^, ^31^ involving a diverse range of participants (*n* = 57). Three 40‐min focus groups were conducted with staff from one optometry practice (4 optometrists, 1 dispensing optician and 1 optical assistant), hospital staff in one eye department (2 nurse practitioners, 1 eye clinic liaison officer and 2 health care assistants) and third sector staff (5 eye clinic liaison officers). Semistructured interviews (*n* = 41) lasting 40–60 min were undertaken with patients (12), carers (4), receptionists/practice managers (3), health board managers (2), optometrists (9), ophthalmologists (3), healthcare support workers/assistants (2), dispensing opticians (1), orthoptists (1), imaging personnel (2), optical assistants (1) and co‐ordinator (1). Patient and carer interviews were conducted first to ensure that the voice of the person receiving eye care was given primacy^32^ by ensuring that the research was grounded in the experiences, perspectives and needs of the patients and their carers. This approach places the patients and carers at the centre of the research process. Interview data were further triangulated with four optometrists' reflective diaries which explored experiences of enhanced optometric services in primary care. All focus group and interview data were recorded within MS Teams, transcribed verbatim and coded within NVivo 12 (Lumivero: lumivero.com/products/nvivo/), leading to the further development of the Programme Theory inducted within the initial Realist Review.

### Health economic evaluation

**FIGURE 1:**
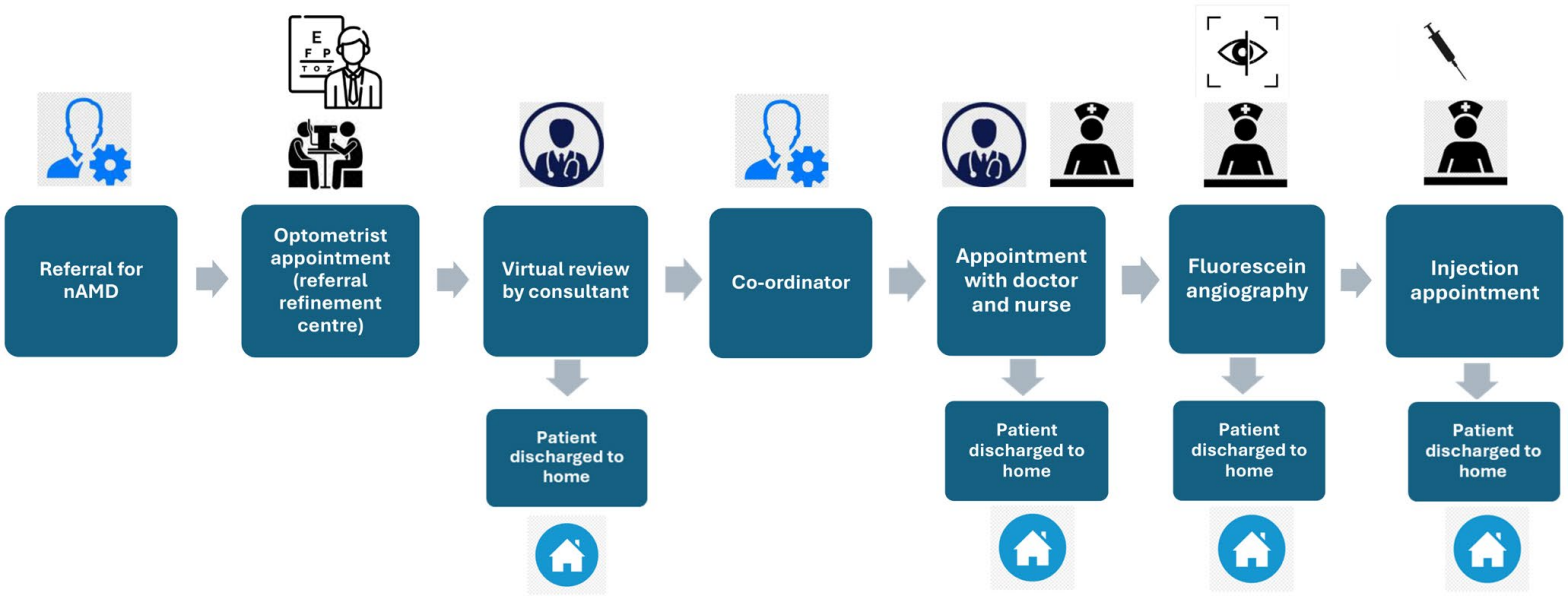
A schematic of the model for the referral refinement virtual review by ophthalmologists (nAMD‐VR) pathway.

A discrete event simulation (DES) model was built based on the three nAMD and four glaucoma pathways. All seven models were constructed using Simul8 (Simul8 Corporation, Professional, version 29, simul8.com).^33^ The models use ‘resources’, in this case members of staff, assigned to ‘events’, for instance an optometrist appointment, in the patient pathway. A pictorial representation of one of the Models is shown in Figure 1. This approach tracks each patient through the pathway, steps through each ‘event’ a person encounters and calculates metrics such as waiting time (in this model, this is the time from when the patient first enters the pathway to the relevant event), time spent in the ‘event’ and number of people waiting in a queue. Simul8 enables incorporation of capacity constraints, staff doing multiple tasks and queueing bottle necks.^34^, ^35^ Each interaction with a health care professional results in costs for the NHS regardless of whether it is a false‐positive referral or true‐positive referral, a query or advice. Interactions were determined from interviews with professionals and have all been costed into the model for each arm of the model.

It was assumed that, for each of the nAMD pathways, patients followed the same sequence of events as outlined in Figure 1, with or without a referral refinement centre and virtual review. All percentages in the model were obtained from audit data for the NHS in Wales. Timings of events in the pathway were also obtained from audit data and interviews with hospital and primary care‐based staff involved in all pathways. All assumptions were verified with clinicians. Six of the DES models were based on routinely collected data, such as audits, and this facilitated analysing future demand in a sensitivity analysis. In the absence of data, a seventh DES model based on the G‐OD pathway assumed similar outcomes to G‐HOVR.

The primary outcome for each of the models was the cost of providing the service for 1 year. For the nAMD models, secondary outcomes included waiting time from initial referral to attend a HES clinic, the number of HES visits that could have been avoided and the percentage of time that the consultant spent on face‐to‐face nAMD appointments. For the glaucoma models, secondary outcomes included the number of consultant appointments and waiting time at the clinic for monitoring.

For the nAMD models, on average, 1300 identical patients were generated to walk through the pathway for 1 year (this number varied annually as unscheduled arrivals follow a Poisson process^36^) based on the estimated number of nAMD‐OD appointments encountered in one health board over 1 year. One HES clinic was run weekly (3.75 h) for all models. In the nAMD‐VR pathway, the consultant was able to perform virtual reviews at any time during the working week outside of clinic time. It was assumed in each face‐to‐face clinic that eight patients were seen, and in each virtual clinic, 20 scans were reviewed (clinic numbers based on data from Wales).

For the glaucoma models, 2880 identical patients were generated, again in keeping with numbers provided by a health board. Six HES clinics were run weekly (3.75 h) for G‐T, with two clinics for G‐VR, G‐HOVR and G‐OD schemes. It was assumed that, in each face‐to‐face clinic, 15 patients were seen and, in each virtual clinic, 20 scans were reviewed (clinic numbers based on data from Wales).

In both nAMD and glaucoma pathways, it was assumed that primary care‐based optometrists could book appointments at any time between Monday and Friday, 09:00–17:00 h. Although some optometry practices were open in the evenings and weekends, there were variations between practices, but all were open 09:00–17:00 h on weekdays. During the interviews with the optometrists, they reported that every effort was made to ensure that appointments occurred for patients who required them within 24 or 48 h at most. At the practices contacted, patients were slotted into clinic between others or at the beginning or end of the day rather than having a set weekly slot. Where a coordinator was present, the model assumed they work Monday–Friday 09:00–17:00 h to book appointments and coordinate the clinic. All other staff were available within the clinic times only.

We used 1‐year time horizons. It is usual to begin to collect results when the DES model is in steady state. Since the HES is under huge pressure, it is impossible to reach steady‐state conditions as the situation is inherently volatile. To mitigate this situation, a 5‐year warm‐up period was used to get closer to steady state.

For the analysis, we observed the flow of resources in the modelled simulation in a steady state over a 12‐month timeline; therefore, no discount rate was applied to the base case analysis. A discount rate of 3.5% was applied to costs in line with NICE recommendations^37^ for time horizons over 12 months considered in the scenario analyses.

Descriptions of the services for nAMD and glaucoma were developed from service operating protocols and interviews with staff. The descriptions outlined the staff and their roles, the tests and scans conducted and time for follow‐up appointments and review of scans. Unit costs for staff time were applied for hospital and primary care‐based services staff.^38^ The NHS reimbursement price^39^ was applied to appointments in hospitals and a cost of £62.74 for appointments in primary care‐based services as this was the average fee at the time.

Sensitivity analyses were undertaken to ensure the model was sufficiently robust to deal with changes in the individual input parameters. Several scenarios were examined to see their impact on outcomes for both costs and waiting times.

### Workforce survey

We worked with Health Education and Innovation Wales and Optometry Wales to develop a workforce survey for optometrists. This Microsoft Forms (Microsoft.com) survey was circulated to all optometrists via NHS Wales Shared Services Partnership and three follow‐up emails were sent. In addition, Optometry Wales and Health Education and Innovation Wales used a link to the survey embedded in newsletters and social media to encourage practitioners to respond. Frequency tables and graphs were constructed to get a picture of the primary care‐based optometry workforce in Wales from 29 April to 1 June 2021. Following on from the survey, Health Education and Innovation Wales kept records of optometrists with higher qualifications, and in May 2023, the updated numbers of optometrists with higher qualifications were requested.

### Ethics

IRAS approval (ID 304550) was obtained for the interviews and focus groups. Other elements of the research were deemed to be service evaluation (ABUHB R&D reference number: SE/1254/21, CTUHB R&D reference number: CT/1449/21 & SB letter of approval 27 May 2021). The workforce survey was conducted in partnership with Health Education and Innovation Wales as part of their workforce planning duties.

## RESULTS

### The eye care PREM

Surveys were received from 802 participants, aged from 28 to 99 years. The sample was fairly equally spread across genders, with 51% female, more respondents accessing glaucoma services (62%) than nAMD (38%) and 64% from the primary care‐based services pathways compared to 36% from hospital. The questionnaire was completed partially by some respondents (*N* = 243), with some questions returning as many as 15% missing values.

The PREM responses showed that, for both HES and primary care‐based services, patients were happy with their experiences. There were no significant differences between the experiences (Pearson's chi‐square, *p* = 0.61) for glaucoma patients attending hospital and community services. However, there was a significant difference between the experiences for AMD patients (Pearson's chi‐square, *p* < 0.0001), with community gaining more responses in the top ‘strongly agree’ category.

### Realist review and evaluation

**FIGURE 2:**
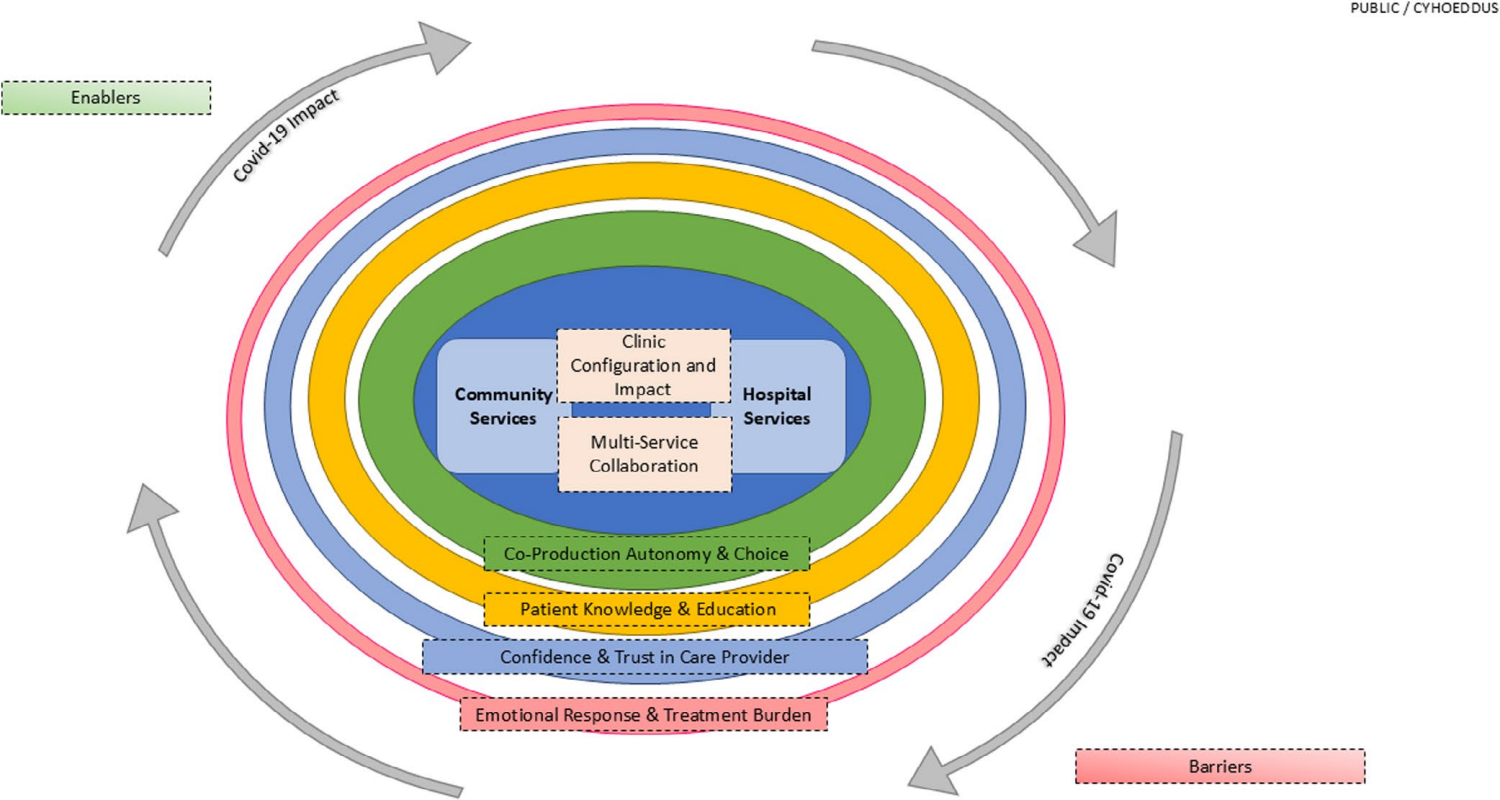
Diagrammatic representation of the Refined Programme Theory developed through three cycles of realist reviews. The mechanisms that influence the implementation and outcomes of the enhanced optometry services are presented and the thickness of each line reflects the amount of evidence underpinning each element.

Through three cycles of realistic review, which included literature reviews, Stakeholder Group input, surveys, interviews, focus groups and reflective diaries, the Refined Programme Theory was developed. This aimed to clarify the mechanisms or themes that influenced the implementation and outcomes of the enhanced optometry services in primary care optometry practices and provide a comprehensive and empirically validated framework for understanding and improving the effectiveness of the services. The Refined Programme Theory is shown in Figure 2. Service location and collaboration between services came out as the themes of greatest influence for these services.

#### Theme: Clinic configuration and impact

Patients perceived primary care‐based services to be more accessible geographically, mitigating treatment and carer burden, and they particularly liked being able to have open communication and flexibility in arranging appointments in practices.

A dedicated primary care‐based service can support patient confidence.

Fine you know, the timing, the organisation there when you go in, they've got it well set out where you do the parameter test, they put the eye drops in… once you are done, the optician or the doctor whoever is there… it's all done down to a fine art. (Patient—primary care based service)

The most notable barriers for patients pertained to the physical inaccessibility of HES services compared with enhanced optometric services, for many patients, primary care‐based services were more physically accessible, requiring reduced travel and carer burden.

The greatest barrier for primary care practitioners was financial; due to the administrative burden and subsequent reduced clinical capacity, primary care clinics experienced a loss of revenue with a fee of only £62.74.

It was found that sustaining an integration between HES and primary care‐based services requires coordination and shared IT protocols with responsive maintenance. A referral coordinator enabled appropriate staffing decisions in primary care‐based services ensuring the number of patients matched the capacity to meet demand. If this role is missing, then the clinical capacity is reduced due to administrative burdens, resulting in increasing patient waits due to inefficient use of primary care‐based services clinics.

What's needed is regional level administrative team to take referrals, then they do the coordinating of sending them out to eligible practices and to hospital, if need be, that's what's missing, that's what's needed in the pathway is proper administration. (Ophthalmologist)

Additionally, practitioners described lack of IT (information technology) support as a barrier—noting issues in accessing patient data, and delays in IT support when systems fail. (Ophthalmologist)

#### Theme: Multi‐service collaboration

It was found that, when interprofessional relationships were not established from the outset, there could be scepticism from HES regarding primary care capabilities. This led to reluctance from HES clinicians to refer their patients into primary care‐based services, resulting in perceived inefficient utilisation of services.

And the one thing I learnt with these schemes as well is that the jeer amongst ophthalmologists is that there's… the optoms will take all our work away, there'll be nothing for us to do, we'll be twiddling our thumbs, we'll be purposeless… (Ophthalmologist)

Furthermore, effective communication between primary and secondary care facilitates trusting reciprocal relationships between professionals, resulted in supporting collaborative service integration. Building this trust and confidence in the abilities of primary care‐based services emerged from effective communication and feedback loops.

so that also was an added complication that you know, it was communication. Communication wasn't there and that caused a difficulty as well for us… but it started moving, got everything down on paper so at least then we had documents to share and discuss with other colleagues which really did help. (Health Board Manager)

Collaborative multiservice partnership was the strongest enabler for practitioners. The effective communication and feedback loops between HES and primary care‐based clinics were highlighted as being of vital importance for effective services. In this evaluation, it was found that when there was adequate mentorship, primary care clinicians felt trusted and supported—this increased their confidence and motivation. Participation in enhanced service schemes also allows optometrists to be exposed to more challenging clinical cases which increases experiential knowledge, along with debriefing and clinical supervision with ophthalmologists. This can create opportunities to use new clinical skills, resulting in enhanced decision‐making and increased motivation.

#### Theme: Co‐production, patient autonomy and choice

Clear communication of information from clinician to patient supports patient understanding of their condition and treatment. If patients receive little explanation, it leads to a poor user experience.

Likewise, a clearly communicated treatment roadmap increases patient agency. This improved communication with patients can result in higher levels of treatment concordance and improved satisfaction.

the lady in ####### said to me ‘this is urgent, we should red flag you about this’, so I said ‘what does that mean’ and she said ‘well they should see you within the next two weeks.’ (Patient)

#### Theme: Patient knowledge and education

In this review, it was found that patient education, knowledge and understanding of eye health can influence their treatment concordance and satisfaction with their care.

So I want to know what's happening, they are my eyes you know… it's not some great secret you know ‘better not tell him this, it might upset him’…so they tell me the result and they said ‘well I think this has changed, this will be referred to in the report’ so I know what's happening… and then I get this letter back saying ‘oh change the drops to something else or’ you know. (Patient)

#### Theme: Confidence and trust in care provider

A patient's confidence and trust in their care provider needs to be cultivated as it can influence their level of engagement with services. The development of such relationships takes time in consultations, in addition to sharing detailed and timely explanations with patients about their condition.

Personally, I tend to take a bit of time where I can, to go over my findings, repeatedly, if need be, so I feel comfortable that they are leaving understanding what has happened at that visit today, a bit about their condition and then what's going to happen at the next appointment… (Patient)

In this evaluation, it was found that prolonged gaps in communication result in patients feeling devalued and losing confidence in their care.

Not very confident… and to be perfectly frank when the results come back after I've had my last of the examination… I might have had the examination in April and generally I get the letter in October because it takes 3 or 4 months for it to be typed…absolutely hopeless. (Patient)

Furthermore, when patients do not receive clear information from primary care‐based clinics, they may lack trust in primary care‐based service practitioners and become reluctant to engage with optometry services.

#### Theme: Emotional response and treatment burden

The evaluation identified that when patients receive bad news such as loss of their driving licence, this manifests as a shocking experience and results in a loss of independence.

Well its really changed my life obviously because I had my own car, I've driven for almost 50 years and that's all gone and I had no idea that this would sort of change so dramatically for me and nothing would be done you know. (Patient)

When clinicians take time to break bad news, this gives people an opportunity to think and process the information and accept the bad news.

They talk to you, they know probably everything about me now, they know I lost my husband, they held my hand when I was crying in there you know…and you need something like that. (Patient)

This review identified conflicting findings with some patients accepting a higher treatment burden in exchange for improved visual acuity, with others preferred less intensive treatment at the cost of lower visual acuity.

#### Theme: Sharing accessible information

To address this knowledge gap, we found evidence that suggested patients often seek clarification from multiple sources, for example, such as online platforms, which vary in accuracy and reliability.

I feel the communication is lacking when you think most people that I know have got a mobile phone … lots of people have internet connection. I think they should use that more as well you know, to offer you information about the complaint you are suffering from as well. (Patient)

When information is provided in a range of modes, patients could more effectively assimilate the information at their own pace; this improved understanding and increased confidence in managing one's condition. The range of requirements also included clinicians providing information at the right time in clinic as per the needs of the patient and in accessible non‐clinical language. This all resulted in patients becoming more informed, resulting in a positive experience of the services.

### Health economic evaluation

The costs, resource use and anticipated waiting times for providing the nAMD and glaucoma clinics in HES and enhanced optometric services are provided in Tables 1 and 2, respectively. The modelled base case results for waiting times were found to match descriptions of real‐life services provided by primary care‐based optometrists, consultant ophthalmologists and managers. Costs are calculated on a cost per clinic basis since the clinic is run regardless of whether a patient attends or not. Glaucoma clinics are less costly than AMD clinics by approximately £600 per clinic (£1200 compared with £1800 per clinic).

**TABLE 1:** Outcomes for neovascular age‐related macular degeneration (nAMD) discrete event simulation (DES) models for 1 year.

	Service pathway		
Outcome	nAMD‐T	nAMD‐VR	nAMD‐OD
Cost to run the service for 1 year	£215,345	£147,560 (£185,681)	£142,009 (£175,130)
Number of optometrist appointments	0	1172	1172
Number of HES appointments	956	221	304
Number of ophthalmology virtual reviews	0	1172	0
Average waiting time for appointment from initial optometrist appointment to review and decision	30 days	4 days	5 days
Number left on waiting list	216	3	3
% time consultant was ‘busy’	57	16	15

*Note*: Primary care optometry pathway with appointment cost £62.74 (and later £91). Average wait time for appointment refers to wait time from referral to discharge (if referral was unnecessary) or to secondary care appointment.

Abbreviations: nAMD‐OD, a primary care optometrist with a Professional Certificate in Medical Retina reviewed the scans and made the decisions; nAMD‐T, traditional treatment in the HES (hospital eye service); nAMD‐VR, virtual review by ophthalmologists.

**TABLE 2:** Outcomes for glaucoma (G) discrete event simulation (DES) models for 1 year.

	Service pathway			
Outcome	G‐T	G‐VR	G‐HOVR	*G‐OD
Cost to run the service for 1 year	£341,929	£317,910 (£403,884)	£303,400 (£389,374)	£282,929 (£368,903)
Number of optometrist appointments	0	3042	3042	3042
Number of HES appointments	1185	415	415	585
Number of ophthalmology virtual reviews	0	3033	3033 (with hospital optometrist)	0
Average wait time for monitoring appointment from requesting	601 days	5 days	5 days	5 days
Number on the waiting list	5691	5	5	5
% time consultant was ‘busy’	48	23	23	22

*Note*: G‐T—traditional pathway with all services in the Hospital Eye Service (HES), G‐VR scans and tests from primary care‐based services reviewed virtually by a consultant ophthalmologist, G‐HOVR—hospital optometrists review the results virtually *G‐OD—optometrists examine the patient and make decisions in primary care practice. *Service modelled, no audit data available. Primary care‐based optometry appointment cost is £62.74 (and later £91). Average wait time for appointment refers to wait time from referral to discharge (if referral was unnecessary) or to clinic appointment.

#### nAMD services

The primary outcome, estimated costs of providing the services for 1 year, was less for both nAMD‐VR and nAMD‐OD than for the nAMD‐T service pathway (Table 1) due to fewer hospital visits (nAMD‐VR *N* = 221, nAMD‐OD *N* = 304, compared with *N* = 956 for nAMD‐T). The waiting times for secondary care appointment or discharge (if no appointment was necessary), accumulated from backlogs generated as the model settled into a steady state over time, were much greater for nAMD‐T, 30 days compared to 4–5 days for those using primary care‐based optometry (Table 1).

Upon completion of the project, the enhanced optometric service fees were raised from £62.74 to £91 per patient as part of the new contract for optometry across Wales. This increase in fee resulted in an overall increase in cost in all pathways incorporating primary care (nAMD‐VR and nAMD‐OD) (Table 1).

#### Glaucoma services

For glaucoma care, the pathways using hospital optometrists and consultants to virtually review the information from primary care‐based services were the costliest (G‐VR and G‐HOVR).

The waiting time for patients from request for a monitoring appointment to the clinic visit was almost 2 years for patients on the G‐T pathway (which is similar to the 100 weeks quoted by an Ophthalmologist). This wait was much longer than the 5 days for services with elements in primary care. Doctors were substantially busier in the G‐T pathway (48%) compared with pathways in which elements were delivered in primary care‐based services (23%, 23% and 22%). All services involving primary care‐based optometrists also freed up substantial numbers of appointments in the HES (Table 2).

The important aspect of this modelling is that it allows a direct like‐for‐like comparison to see where marginal change occurs when patients move into alternative pathways and where the benefits lie. Importantly, the plausibility of the modelling was investigated, as well as how changing parameters may change the resource flow and the costs by creating different scenarios and undertaking sensitivity analysis (provided in Table S1). Even when ‘pressure testing’ the modelling in this way, the results suggest the beneficial changes in both capacities gained in the HES, the reduced waiting times for nAMD diagnosis and monitoring appointments for glaucoma, are maintained. For some services in the primary care setting, it was estimated that there were small additional costs for providing the service; however, there are always gains in waiting list reduction and capacity gains in HES.

Applying the enhanced optometric services fee uplift to the glaucoma pathways resulted in an increase in all pathways involving primary care elements (G‐VR, G‐HOVR and G‐OD) (Table 2).

### Workforce

There were 460 respondents to the workforce survey including 439 with full information. Respondents were predominately female (57%) and White (86%). Most (62%) studied their undergraduate degree at a Welsh University. The aim of the analysis was to ascertain whether there was sufficient optometry workforce in Wales with the required qualifications for roll‐out of the enhanced optometric services for glaucoma and nAMD.

For nAMD‐VR and nAMD‐OD, optometrists require the professional certificate in medical retina (*n* = 71). For G‐VR, they require a professional certificate in glaucoma (*n* = 105), and for G‐HOVR and G‐OD, the higher certificate in glaucoma (*n* = 13). Many more optometrists were studying for a range of relevant qualifications at the time of the survey (*n* = 106) and should have completed by the time of this publication.

Personal written correspondence from the Head of Optometry, Health Education and Innovation Wales (February 2024) reported that the numbers of optometrists with higher qualifications had increased substantially in just 2 years. By September 2023, there were 137 optometrists with professional certificate in medical retina (93% increase) and a further 16 in training, 164 with professional certificate in glaucoma (56% increase) and a further 18 in training and 38 with a higher professional certificate in glaucoma (192% increase) and a further 18 in training.

## DISCUSSION

The simulation modelling, designed and informed by the literature, stakeholders, patient and staff interviews, and populated with data from the clinics and expert informants, suggests that integrating primary care‐based optometrists with higher qualifications in the referral refinement for patients with nAMD and in monitoring patients with glaucoma will dramatically reduce waiting times, and also release consultant ophthalmologist time.

In the different analyses, these findings indicate that deploying enhanced optometric services in primary care will reduce the wait time for people with suspected nAMD to 4–5 days and a glaucoma monitoring visit can be achieved within 5 days of the target date. Utilising primary care‐based services over the course of a year reduces the accumulating number of people on the waiting list to three at the end of the year, compared with 216 in a HES for nAMD and five people compared to 5691 for glaucoma. The modelling suggests that such shifts of activities within the pathway, over time, could help reduce long waiting times, clear backlogs and importantly, with patients seen promptly, reduce the risk of people losing sight because of delays.

The reduction of consultant ophthalmologist time in service provision from 57% to 15%–16% for nAMD services and from 48% to 22%–23% for glaucoma services represents a considerable amount of freed resource. Consultant ophthalmologists are the most highly trained and expensive health professionals in the eye care pathway. This modelling suggests that greater use of primary care‐based optometrists in suspected nAMD and glaucoma monitoring pathways could free up ophthalmologist time to see patients that only they can manage and for them to undertake high value or more specialist activities within the HES. This will result in hundreds of additional outpatient appointments that could be used for patients that only should be seen by consultant ophthalmologists. Importantly, this could help prevent sight loss by ensuring patients are seen in a timely manner.

Importantly, for patients themselves, a timely appointment for suspected nAMD and for monitoring glaucoma may reduce their concerns as well as providing more swift reassurance about their eye condition or its management. Through the Realist Review and Evaluation, it was found that patients in both HES and primary care‐based services value receiving the right service in the right place and at the right time. Patients perceived primary care‐based clinics to be more accessible geographically and being able to negotiate appointment times was also important. Others have found that this can lead to greater engagement with eye care services.^21^ Using the Eye Care PREM, it was found that patients using all pathways of glaucoma and nAMD services were satisfied, which is reassuring.

The greatest barrier for optometrists providing these services in primary care was financial and practitioners reported that they experienced a loss in revenue when the fee was only £62.74. Since study completion, the contract for optometrists across Wales has changed and a WGOS4 fee to cover this type of service for optometrists is £91 for referral refinement and monitoring in primary care‐based services.

The new fee caused the nAMD‐VR pathway to increase in cost from £147,560 to £180,681 per year and the nAMD‐OD pathway to increase from £142,009 to £175,130. These new costs are still cheaper than the traditional HES pathway (£215,345) for nAMD patients. For the glaucoma pathways, an increase was again seen in all primary care‐based pathways, with G‐VR costing £403,884 (compared to £317,910), G‐HOVR costing £389,374 (compared to £303,400) and G‐OD costing £368,903 (compared to £282,929). The G‐OD pathway, in which optometrists with higher qualifications make decisions, costed with the higher fee of £91 (£368,902) was the cheapest model involving primary care and is marginally more costly compared with the traditional HES pathway (£341,929). In England, the tariff for a hospital consultant led outpatient appointment for ophthalmology was £160 for first attendance and £78 for a follow‐up.^40^

The NICE compliant pathways utilising optometrists to assess patients and make decisions, rather than relying on hospital optometrists or ophthalmologists to virtually review their work, freed up considerable hospital resources which could be better used for those patients that can only be managed in hospital. All the estimates suggested that integrating a primary care optometrist with higher qualifications into the pathway to make more decisions in primary care, and using the new fee being introduced in Wales for the WGOS4 pathway, will use approximately the same budget. However, if implemented across Wales, these changes will significantly free up HES resources and consultant ophthalmologist time.

When Sharma et al.^41^ compared hospital‐based glaucoma clinics to community‐based clinics, the community services were found to be more expensive due to higher overhead costs, although the authors reported this could be improved by increasing the number of patients seen in the community. It may be that the community services in Wales had a greater throughput of patients, or the overheads may be different in alternative areas as the Sharma et al.s^41^ cost analysis involved London‐based clinics.

Eighteen months after the end of data collection, all three health boards reported that no safety incidents had been reported relating to the enhanced optometry service pathways during the time of the study.

Changing the number of patients seen within each HES clinic does not have an impact on cost (all clinics run regardless of the number of patients); however, it does mean that patients within the traditional pathway experience longer or shorter waiting times. Changing the grade of ophthalmologist or hospital optometrists seeing patients or doing the virtual reviews could reduce the cost. However, the capacity and number of appointments released in hospitals does not change. Likewise, increasing the number of hospital clinics or staff would reduce waiting times, but interviews with staff and the present modelling suggested this was not usually possible and/or was not sustainable.

### Factors that are enablers and barriers for the development of primary care‐based enhanced optometric services

Central to good patient ‘experience’ was clear communication of information and providing good patient education about eye health and the service pathway. These themes were recurring and overlapping findings in the Realist Review. When services are moved to primary care‐based services from the HES, ensuring that accessible patient information about the service pathways and conditions is available in a range of formats (leaflets and websites) will enable patients to feel empowered,^42^ improve treatment concordance and address access disparities.^43^ When patients perceive their care provider as knowledgeable, their trust in them increases, and this can influence engagement with services and treatment concordance.^44^

The findings of the Realist Review and Evaluation suggest that changing service pathways will work for all eye care professionals and the services they work within, if attention is paid to the roles of both the coordinators and administration staff who ‘manage’ the lists in the primary care‐based practices and HES. Their clear and constant communication ensures that there is continuity of service and primary care optometry can plan effectively to meet the pathway requirements.

It takes time to establish and maintain interprofessional communication, especially between optometrists and ophthalmologists, and this is essential for this to work effectively, building trust and confidence. IT, shared records, information sharing protocols and responsive maintenance are essential to ensure that services can share appropriate information which will meet the needs of patients.

There is no doubt that integrating secondary and primary care services is challenging NHS Services. A systematic review of integrated models of health care delivered at the primary–secondary interface outlined very similar themes for effectiveness found in this study: (1) interdisciplinary teamwork; (2) communication/information exchange; (3) shared pathways; (4) training and education; (5) access and acceptability for patients and (6) a viable funding model.^45^

### Capacity and qualifications of optometrists in Wales

Crucial to implementation or substantial expansion of the proposed primary care services is the capacity of the optometrists in the locations required. That means that the capability (the training and equipment needed) to provide the service must be in place.

In order to change to a primary care‐based service, a critical mass of optometrists with higher level training must be available. To investigate these factors and to estimate the potential to deliver the primary care‐based service in Wales, the Optometry Wales Workforce survey and routinely collected data from Health Education and Innovation Wales were utilised. In addition, interviews with optometrists were undertaken.

Overall, the optometrist workforce in Wales has considerable capacity in all the health boards to support both the nAMD referral refinement and the glaucoma monitoring services; the training and equipment to deliver the service is also in place, more optometrists are being trained and the current workforce is willing. In 2021, our survey found there were 13 optometrists with a higher certificate in glaucoma, and this is very similar to the 16 reported from the College of Optometrists data.^46^ By 2023, Health Education and Innovation Wales found this had increased dramatically, with 56 optometrists studying for or having completed the higher certificate in glaucoma. This increase in uptake highlights the appetite of optometrists to take on this work.

### Limitations of the research

While the data that drive the results presented and discussed in this report are based on robust sources, the data are mainly routinely collected service data, not clinical trial data, that have been synthesised using economic modelling techniques. While we have endeavoured to develop a model structure that represents reality, informed by our stakeholders, it is inevitably a simplification, but we have taken care to be sure that the base case is conservative in its approach, and undertake scenario and sensitivity analysis to explore what happens when the inputs are changed.

The method of PREM collection was co‐produced with the Stakeholder Advisory Group, there was engagement with service leaders and those involved in implementation and multiple methods to respond were provided.^47^ Although the large number of responses received across primary and secondary care makes the results useful, it is difficult to calculate the response rate as it was unknown how many surveys were given and the survey was available online as well as in printed format. Likewise, the accuracy of the NHS Wales Shared Services Partnership email list at that time was not known; hence, there is no way of knowing how many people received the survey, and thus, it is not possible to calculate the response rate.

This research used primary care‐based services already operating in three health boards. These areas were predominantly urban and the primary care services were most often near the HES sites. Much of Wales is rural and optometry practices are frequently a long way from HES. Switching to a primary care service in these areas may improve the value of the service and have additional benefits for the net zero healthcare agenda through greater use of more locally accessible services. We believe these estimates are robust and applicable across Wales. It is difficult to know whether the results can be generalised beyond Wales and further research is needed to understand if the pathways and benefits demonstrated in this study could be replicated elsewhere.

## CONCLUSIONS

These results powerfully suggest significant benefits for the NHS in Wales and for patients if a shift to primary care‐based services is implemented for those attending for preliminary diagnosis of nAMD or glaucoma monitoring services. The benefits will be felt in all parts of the pathway of care, but most importantly the benefits will be tangible for patients, who will not be waiting for an appointment in the HES for so long that their sight is (further) threatened. These results reassuringly suggest that patient experience in primary care‐based services is at least as good and sometimes better than if their care was mainly in HES.

We are confident that a shift of referral refinement services for nAMD, and monitoring services for glaucoma, into primary care using optometrists with higher professional qualifications, will dramatically reduce waiting times and waiting lists in the HES and release meaningful capacity in the HES including consultant ophthalmologist time. Clear patient communication about eye health and the service pathway, ensuring appropriate coordinator and administration staff who ‘manage’ the lists and supporting systems and processes that facilitate good interprofessional communication between optometrists and ophthalmologists, are crucial to the success of this change.

## AUTHOR CONTRIBUTIONS

**Barbara Ryan:** Conceptualization (equal); funding acquisition (lead); investigation (equal); methodology (equal); project administration (equal); resources (equal); writing – original draft (lead); writing – review and editing (equal). **Mari Jones:** Conceptualization (supporting); data curation (equal); formal analysis (equal); investigation (equal); methodology (equal); validation (equal); visualization (equal); writing – original draft (supporting); writing – review and editing (equal). **Pippa Anderson:** Conceptualization (equal); funding acquisition (equal); methodology (equal); visualization (supporting); writing – original draft (supporting); writing – review and editing (equal). **Rhiannon Reynolds:** Conceptualization (supporting); funding acquisition (supporting); methodology (supporting); resources (equal); writing – review and editing (equal). **Rebecca E. M. Nicholls:** Data curation (supporting); formal analysis (supporting); investigation (supporting); methodology (supporting); visualization (supporting); writing – review and editing (supporting). **Katherine Cullen:** Data curation (supporting); formal analysis (supporting); investigation (supporting); methodology (supporting); validation (supporting); visualization (supporting); writing – review and editing (supporting). **Mark Davies:** Data curation (supporting); formal analysis (supporting); investigation (supporting); supervision (equal); visualization (equal); writing – review and editing (supporting). **Rachel North:** Conceptualization (supporting); funding acquisition (supporting); investigation (supporting); methodology (supporting); project administration (supporting); resources (supporting); writing – original draft (supporting); writing – review and editing (supporting). **Bablin Molik:** Conceptualization (supporting); funding acquisition (supporting); methodology (supporting); resources (supporting); writing – review and editing (supporting). **Carolyn Wallace:** Conceptualization (equal); data curation (equal); formal analysis (equal); funding acquisition (equal); investigation (equal); methodology (supporting); supervision (equal); visualization (supporting); writing – original draft (supporting); writing – review and editing (equal).

## CONFLICT OF INTEREST STATEMENT

The authors declare no conflicts of interest.

## FUNDING INFORMATION

This research was funded by Health and Care Research Wales, Research for Patient Benefit‐19‐1639.

## Supporting information


Table S1.

Supplementary S2.

## References

[1] Quartilho A , Simkiss P , Zekite A , Xing W , Wormald R , Bunce C . Leading causes of certifiable visual loss in England and Wales during the year ending 31 March 2013. Eye. 2016;30:602–607. 26821759 10.1038/eye.2015.288 PMC5108547

[2] StatWales . Outpatient attendances by treatment function [cited 2023 Aug 20]. Available from: https://statswales.gov.wales/Catalogue/Health‐and‐Social‐Care/NHS‐Hospital‐Activity/Outpatient‐Activity/outpatient‐attendances‐by‐treatment‐function

[3] NHS England Digital . Hospital outpatient activity 2022–23—NHS digital [cited 2023 Feb 9].

[4] The way forward. 2017. London: Royal College of Ophthalmologists [cited 2024 Feb 9]. Available from: https://www.rcophth.ac.uk/resources‐listing/the‐way‐forward/

[5] Welsh Government . Eye care measures for NHS outpatients: August 2024 [cited 2024 Oct 5]. Available from: https://www.gov.wales/eye‐care‐measures‐nhs‐outpatients‐august‐2024‐html

[6] Foot B , MacEwen C . Surveillance of sight loss due to delay in ophthalmic treatment or review: frequency, cause and outcome. Eye. 2017;31:771–775. 28128796 10.1038/eye.2017.1 PMC5437335

[7] Gale RP , Mahmood S , Devonport H , Patel PJ , Ross AH , Walters G , et al. Action on neovascular age‐related macular degeneration (nAMD): recommendations for management and service provision in the UK hospital eye service. Eye. 2019;33(Suppl 1):1–21. 10.1038/s41433-018-0300-3 PMC6474281 30926932

[8] Karthikeyan A , Lee CN , Myneni J , Harthan S , Bragg K , Bentley S , et al. Three‐year outcomes of an optometrist‐led virtual clinic for new glaucoma referrals. Ophthalmic Physiol Opt. 2023;43:760–770. 36930523 10.1111/opo.13124

[9] NICE . Glaucoma: diagnosis and management [NG81]. 2022 [cited 2024 Feb 9]. Available from: https://www.nice.org.UK/guidance/ng81

[10] NICE . Age‐related macular degeneration NICE guideline [NG82]. 2018 [cited 2024 Feb 9]. Available from: https://www.nice.org.UK/guidance/ng82

[11] The General Ophthalmic Services Contracts Regulations 2008. UK Statutory Instruments. 2008. No. 1185 PART 5 Regulation 13.

[12] The National Health Service (Ophthalmic Services) (Wales) Regulations 2023. Wales Statutory Instruments No. 1053 (W. 179). 2023 [cited 2024 Oct 5]. Available from: https://www.legislation.gov.uk/wsi/2023/1053/made

[13] Welsh Government 2021 . NHS Wales Eye Health Care: future approach for optometry services [cited 2024 Oct 5]. Available from: https://www.gov.wales/nhs‐wales‐eye‐health‐care‐future‐approach‐optometry‐services

[14] Baker H , Ratnarajan G , Harper RA , Edgar DF , Lawrenson JG . Effectiveness of UK optometric enhanced eye care services: a realist review of the literature. Ophthalmic Physiol Opt. 2016;36:545–557. 27580754 10.1111/opo.12312

[15] Mason T , Jones C , Sutton M , Konstantakopoulou E , Edgar DF , Harper RA , et al. Retrospective economic analysis of the transfer of services from hospitals to the community: an application to an enhanced eye care service. BMJ Open. 2017;7:e014089. 10.1136/bmjopen-2016-014089 PMC5541458 28698317

[16] Reeves BC , Scott LJ , Taylor J , Harding SP , Peto T , Muldrew A , et al. Effectiveness of Community versus Hospital Eye Service follow‐up for patients with neovascular age‐related macular degeneration with quiescent disease (ECHoES): a virtual non‐inferiority trial. BMJ Open. 2016;6:e010685. 10.1136/bmjopen-2015-010685 PMC4947830 27401357

[17] Violato M , Dakin H , Chakravarthy U , Reeves BC , Peto T , Hogg RE , et al. Cost‐Effectiveness of Community versus Hospital Eye Service follow‐up for patients with quiescent treated age‐related macular degeneration alongside the ECHoES randomised trial. BMJ Open. 2016;6:e011121. 10.1136/bmjopen-2016-011121 PMC5093395 27797985

[18] Chakravarthy U , Harding SP , Rogers CA , Downes S , Lotery AJ , Dakin HA , et al. A randomised controlled trial to assess the clinical effectiveness and cost‐effectiveness of alternative treatments to inhibit VEGF in age‐related choroidal neovascularisation (IVAN). Health Technol Assess. 2015;19:1–298. 10.3310/hta19780 PMC4781416 26445075

[19] Holdsworth E , Datta J , Marks D , Kuper H , Lee H , Leamon S , et al. A mixed‐methods evaluation of a community‐based glaucoma check service in Hackney, London, UK. Ophthalmic Epidemiol. 2017;24:248–256. 28287859 10.1080/09286586.2016.1272702

[20] Leal J , Luengo‐Fernandez R , Stratton IM , Dale A , Ivanova K , Scanlon PH . Cost‐effectiveness of digital surveillance clinics with optical coherence tomography versus hospital eye service follow‐up for patients with screen‐positive maculopathy. Eye. 2019;33:640–647. 30504828 10.1038/s41433-018-0297-7 PMC6461849

[21] Swystun AG , Davey CJ . A needs assessment for a minor eye condition service within Leeds, Bradford and Airedale, UK. BMC Health Serv Res. 2019;19. 10.1186/s12913-019-4448-8 PMC6716842 31464616

[22] North R , Anderson P , Harris S , Kenkre J , Wallace S , Wallace C . Wet age related macular degeneration services in the community: a pathfinder evaluation‐final report. Cardiff University; 2019 [cited 2024 Oct 5]. Available from: https://orca.cardiff.ac.uk/id/eprint/135534

[23] The College of Optometrists Higher Professional Qualifications [cited 2024 Feb 9]. Available from: https://www.college‐optometrists.org/professional‐development/further‐qualifications/higher‐qualifications

[24] The Royal College of Ophthalmologists , The College of Optometrists . Designing Glaucoma Care Pathways using GLAUC‐STRAT‐FAST Designing‐Glaucoma‐Care‐Pathways‐using‐GLAUC‐STRAT‐FAST. 2022 [cited 2024 Oct 5]. Available from: https://www.rcophth.ac.uk/wp‐content/uploads/2022/04/Designing‐Glaucoma‐Care‐Pathways‐using‐GLAUC‐STRAT‐FAST.pdf

[25] Wong G , Greenhalgh T , Westhorp G , Buckingham J , Pawson R . RAMESES publication standards: realist syntheses. BMC Med. 2013;11:1–4. 10.1186/1741-7015-11-21 23360677 PMC3558331

[26] Wong G , Westhorp G , Pawson R , Greenhalgh T . Realist synthesis. RAMESES training materials. London: The RAMESES Project; 2013.

[27] Marchal B , Van Belle S , Van Olmen J , Hoerée T , Kegels G . Is realist evaluation keeping its promise? A review of published empirical studies in the field of health systems research. Evaluation. 2012;18:192–212.

[28] Pawson R . Evidence‐based policy: a realist perspective. London: Sage; 2006.

[29] Pawson R , Tilley N . Realistic evaluation. New York: Sage; 1997.

[30] Emmel N . Sampling and choosing cases in qualitative research: a realist approach. London: Sage; 2013.

[31] Manzano A . The craft of interviewing in realist evaluation. Evaluation. 2016;22:342–360.

[32] WHO . Framework on integrated people‐centred health services. 2016 [cited 2024 Feb 9]. Available from: https://apps.who.int/gb/ebwha/pdf_files/WHA69/A69_39‐en.pdf?ua=1&ua=1

[33] Vázquez‐Serrano JI , Peimbert‐García RE , Cárdenas‐Barrón LE . Discrete‐event simulation modeling in healthcare: a comprehensive review. Int J Environ Res Public Health. 2021;18:12262. 10.3390/ijerph182212262 34832016 PMC8625660

[34] Standfield L , Comans T , Scuffham P . Markov modeling and discrete event simulation in health care: a systematic comparison. Int J Technol Assess Health Care. 2014;30:165–172. 24774101 10.1017/S0266462314000117

[35] Pesonen M , Kankaanpää E , Vottonen P . Cost‐effectiveness of dexamethasone and triamcinolone for the treatment of diabetic macular oedema in Finland: a Markov‐model. Acta Ophthalmol. 2021;99:e1146–e1153. 33421332 10.1111/aos.14745 PMC8597173

[36] Peter PO , Sivasamy R . Queueing theory techniques and its real applications to health care systems–outpatient visits. Int J Healthc Manag. 2019;14:114–122.

[37] NICE . Developing NICE guidelines: the manual [PMG20]. 2014 [cited 2024 Feb 9]. Available from: https://www.nice.org.UK/process/pmg20/chapter/introduction

[38] Jones KC , Burns A . Unit costs of health and social care. 2021 [cited 2024 Oct 5]. Available from: https://kar.kent.ac.uk/id/eprint/92342

[39] NHS England . National cost collection data publication. 2019/20 [cited 2024 Feb 9]. Available from: https://www.England.nhs.UK/publication/2019‐20‐national‐cost‐collection‐data‐publication/

[40] NHS England . National tariff payment system documents, annexes and supporting documents 2022/23. 2022 [cited 2024 Feb 9]. Available from: https://www.england.nhs.uk/publication/national‐tariff‐payment‐system‐documents‐annexes‐and‐supporting‐documents/

[41] Sharma A , Jofre‐Bonet M , Panca M , Lawrenson JG , Murdoch I . An economic comparison of hospital‐based and community‐based glaucoma clinics. Eye. 2012;26:967–971. 22562188 10.1038/eye.2012.73 PMC3396173

[42] Goyal A , Richards C , Patel V , Syeda S , Guest JM , Freedman RL , et al. The Vision Detroit Project: visual burden, barriers, and access to eye care in an urban setting. Ophthalmic Epidemiol. 2022;29:13–24. 33576279 10.1080/09286586.2021.1884264

[43] Lu TC , Angell B , Dunn H , Ford B , White A , Keay L . Determining patient preferences in a glaucoma service: a discrete choice experiment. Clin Exp Ophthalmol. 2019;47:1146–1155. 31397968 10.1111/ceo.13606

[44] Osborn K , Bradley J , Knox E , Leighton P . What matters to patients? A thematic analysis of patient information and support needs. Eye. 2020;34:103–115. 31719673 10.1038/s41433-019-0644-3 PMC7002456

[45] Mitchell GK , Burridge L , Zhang J , Donald M , Scott IA , Dart J , et al. Systematic review of integrated models of health care delivered at the primary–secondary interface: how effective is it and what determines effectiveness? Aust J Prim Health. 2015;21:391–408. 26329878 10.1071/PY14172

[46] Harper RA , Gunn PJ , Spry PG , Fenerty CH , Crabb DP , Bowen M . Transforming glaucoma care pathways: current glaucoma accreditation in UK optometry. Eye. 2022;36:676–678. 34702971 10.1038/s41433-021-01820-7 PMC8546781

[47] Benson T . Why it is hard to use PROMs and PREMs in routine health and care. BMJ Open Qual. 2023;12:e002516. 10.1136/bmjoq-2023-002516 PMC10749067 38135303

