# Supplementary material for: Hospital to community in Wales: What is the value of optometrists playing a greater role in managing neovascular AMD and glaucoma in primary care?
Source: Ophthalmic Physiol Opt. 2024 Oct 10;45(1):280–93. doi: 10.1111/opo.13397 (PMC11629850; doi:10.1111/opo.13397)
Supplement: Supplementary file 1 — Supplementary file (DOCX 16.4 KB) [file 44402_2025_4501025_MOESM1_ESM.docx]

**Supplementary materials**

Table S1: Examples of scenario and sensitivity analyses undertaken for nAMD service.

|  | **Cost** | | | **Number of secondary care appointments** | | |
| --- | --- | --- | --- | --- | --- | --- |
| Sensitivity | **nAMD-T** | **nAMD-VR** | **nAMD-OD** | **nAMD-T** | **nAMD-VR** | **nAMD-OD** |
| Base case | £215,345 | £147,560 | £142,009 | 956 | 221 | 304 |
| Change optometrist fee – £91 | £215,345 | £215,895 | £210,218. | 956 | 221 | 304 |
| Change staff grade (consultant to registrar) | £136,954 | £129,454 | £117,082 | 956 | 221 | 304 |
| Change staff grade (consultant to associate specialist) | £213,890 | £147,224 | £141,547 | 956 | 221 | 304 |
| Change percentage of optometrist who discharge directly - to 81% (the same as VR* consultant) | £215,345 | £127,560 | £121,601 | 956 | 221 | 213 |
| Change percentage of VR* who discharge directly - to 73% (the same as optometrist) | £215,345 | £169,455 | £142,009 | 956 | 318 | 304 |
| *VR = Virtual review | | | | | | |

Table S2: Examples of scenario and sensitivity analyses undertaken for glaucoma monitoring service.

|  | **Cost** | | | | **Number of secondary care appointments** | | | |
| --- | --- | --- | --- | --- | --- | --- | --- | --- |
| Sensitivity | **G-T** | **G-VR** | **G-HOVR** | ***G-OD** | **G-T** | **G-VR** | **G-HOVR** | ***G-OD** |
| Base case | £341,929 | £317,910 | £303,400 | £282,929 | 1185 | 415 | 415 | 585 |
| Change optometrist fee – £91 | £341,929 | £403,884 | £389,374 | £368,903 | 1185 | 415 | 415 | 585 |
| Change staff grade (consultant to registrar) | £258,700 | £295,502 | £280,992 | £260,521 | 1185 | 415 | 415 | 585 |
| Change staff grade (consultant to associate specialist) | £338,130 | £316,887 | £302,377 | £281,906 | 1185 | 415 | 415 | 585 |
