# Supplementary material for: Hospital to community in Wales: What is the value of optometrists playing a greater role in managing neovascular AMD and glaucoma in primary care?
Source: Ophthalmic Physiol Opt. 2024 Oct 10;45(1):280–93. doi: 10.1111/opo.13397 (PMC11629850; doi:10.1111/opo.13397)
Supplement: Supplementary file 2 — Supplementary file (PDF 282 KB) [file 44402_2025_4501025_MOESM2_ESM.pdf]

Bwrdd Iechyd Prifysgol Aneurin Bevan University Health Board

Ymchwil Iechyd a Gofal Cymru / Health and Care Research Wales

Hospital to Community: Eye care services for glaucoma & wet age-related macular degeneration.

Thank you for attending your appointment today.

- **Would you be willing to help us by commenting on the service you have received? It will take about 5 minutes.**
- **Your participation is voluntary, and your answers are anonymous.**
- **Your answers will help us improve the service in the future for you and other patients.**

Do you agree to the above terms? By ticking Yes, you consent that you are willing to answer the questions in this survey.

YES		NO	

Do you consent with your personal data being processed as described above? You must answer Yes in order to take the survey.

YES		NO	

**Date:**
**Name of Practice or Hospital:**
**Are you attending for glaucoma or macular degeneration?**<br>**Glaucoma** ☐<br>**Macular degeneration** ☐

THIS SURVEY SHOULD BE COMPLETED AND PLACED IN THE LOCKED BOX AFTER YOUR APPOINTMENT. ***Thank you***

There are 4 sections to this survey. Sections 1 & 2 can be completed before your appointment and Sections 3 & 4 need to be completed after your appointment. If you are unable to complete the survey during your visit we will provide a stamped addressed envelope for you to return it.

1. About you

1.1 Are you?

Please tick in the box

Female	

Male	

1.2 How old are you?

Please write in the box

Years	

1.3 What are the first four digits of your postcode?

Please write in the box

1.4 How long did it take to get to your appointment today?

Please write in the box

Hours	
Minutes	

2. Your appointment today

2.1 From the time I realised I needed to use this service the time I waited for the appointment was about right

Please tick in the box

Strongly agree	
Agree	
Neither agree nor disagree	
Disagree	
Strongly disagree	

2.2 Did you have to take time off work to attend this appointment today?

Please tick in the box

Yes		No	

2.3 If yes, how much time did you take off?

Please tick in the box

Up to ½ day (3-4 hrs)		Whole day (7-8 hrs)	

2.4 If you were accompanied to your appointment (e.g. by spouse or a carer) did they need to take time off work to attend?

Please tick in the box

Yes		No	

2.5 If yes, how much time did they take off?

Please tick in the box

Up to ½ day (3-4 hrs)		Whole day (7-8 hrs)	

2.6 If you have a health condition or disability that affects your mobility, you can apply for a parking permit, often called a 'Blue Badge' from your local authority. Do you have a blue badge?

Please tick in the box

**Yes**		**No**		**Do not know**	

2.7 Please tick the list of aids that you used to get to your appointment.

**Walking stick**	
**Crutch/es**	
**Manual Wheelchair**	
**Electric Wheelchair**	
**Electric scooter**	
**Mobility cane**	
**Low vision aids**	
**Other**	

If "other" please specify, below

2.8 What transport did you use to travel to your appointment today?

Please tick in the box

**Bus**	
**Train**	
**Car**	
**Taxi**	
**Voluntary hospital car service**	
**Hospital ambulance service**	
**Other**	

If "other" please specify, below

2.9 If you drove your car or were driven how many miles did you drive each way?

Please write in the box

Miles each way

2.10 How much did you pay for car parking?

Please write in the box.

Please put 0 if you did not pay.

£

2.11 If you took public transport, how much did you pay in total? Please put 0 if you have a bus pass and did not have to pay.

Please write in the box

£

In total

2.12 Did you combine your visit to the eyecare appointment with any other activities?

Please write out what you did below

3. Your experience of the appointment

Please tick the answer that best fits your experience at the practice TODAY.

3.1 "The eye care service location/premises are accessible"
Please tick in the box

Strongly agree	
Agree	
Neither agree nor disagree	
Disagree	
Strongly disagree	

3.2 "The environment is clean and comfortable"
Please tick in the box

Strongly agree	
Agree	
Neither agree nor disagree	
Disagree	
Strongly disagree	

3.3 "The service provided at the reception was good"
Please tick in the box

Strongly agree	
Agree	
Neither agree nor disagree	
Disagree	
Strongly disagree	

3.4 “I was confident with the assessment provided by the eye care practitioners”
Please tick in the box

**Strongly agree**	
**Agree**	
**Neither agree nor disagree**	
**Disagree**	
**Strongly disagree**	

3.5 “I was treated with dignity and respect by the eye care practitioner(s)”
Please tick in the box

**Strongly agree**	
**Agree**	
**Neither agree nor disagree**	
**Disagree**	
**Strongly disagree**	

3.6 “If I asked for assistance, I received it”
Please tick in the box

**Strongly agree**	
**Agree**	
**Neither agree nor disagree**	
**Disagree**	
**Strongly disagree**	
**Not applicable**	

3.7 "I had enough time with the eye care practitioners today"
Please tick in the box

**Strongly agree**	
**Agree**	
**Neither agree nor disagree**	
**Disagree**	
**Strongly disagree**	

3.8 "I felt the eye care practitioners understood my condition"
Please tick in the box

**Strongly agree**	
**Agree**	
**Neither agree nor disagree**	
**Disagree**	
**Strongly disagree**	

3.9 "I felt well cared for during the assessment today"
Please tick in the box

**Strongly agree**	
**Agree**	
**Neither agree nor disagree**	
**Disagree**	
**Strongly disagree**	

3.10 "I felt I could ask the eye care practitioners questions and my concerns were listened to"
Please tick in the box

**Strongly agree**	
**Agree**	
**Neither agree nor disagree**	
**Disagree**	
**Strongly disagree**	

3.11 "Things were explained to me in a way I could understand"
Please tick in the box

**Strongly agree**	
**Agree**	
**Neither agree nor disagree**	
**Disagree**	
**Strongly disagree**	

3.12 "I feel I understand what is happened with my care"
Please tick in the box

**Strongly agree**	
**Agree**	
**Neither agree nor disagree**	
**Disagree**	
**Strongly disagree**	

3.13 "I feel I have been given enough information about what will happen next"
Please tick in the box

**Strongly agree**	
**Agree**	
**Neither agree nor disagree**	
**Disagree**	
**Strongly disagree**	

3.14 "I am involved (as much as I would like to be) with decisions in my care"
Please tick in the box

**Strongly agree**	
**Agree**	
**Neither agree nor disagree**	
**Disagree**	
**Strongly disagree**	

4. Your experiences of the appointment – COVID-19 safety measures

4.1 "There were adequate hand sanitising facilities in place in the eye care clinic"
Please tick in the box

**Strongly agree**	
**Agree**	
**Neither agree nor disagree**	
**Disagree**	
**Strongly disagree**	

4.2 "All clinic staff wore face coverings in line with government guidelines"
Please tick in the box

**Strongly agree**	
**Agree**	
**Neither agree nor disagree**	
**Disagree**	
**Strongly disagree**	

4.3 "There were social distancing measures, such as signage in place"
Please tick in the box

**Strongly agree**	
**Agree**	
**Neither agree nor disagree**	
**Disagree**	
**Strongly disagree**	

4.4 "I felt safe at my appointment with the COVID-19 safety measures in place"
Please tick in the box

**Strongly agree**	
**Agree**	
**Neither agree nor disagree**	
**Disagree**	
**Strongly disagree**	

If you have any further comments about your appointment today, please write them in the space below (continue over page if necessary).

Thank you for completing this survey

If you have any questions please contact:

Dr Bablin Molik, Sight Cymru
Tel: 01495 763650
Email: bablin.molik@sightcymru.org.uk

Lead Researcher & Team: Prof Barbara Ryan, ABUHB, Dr Pippa Anderson & Dr Mari Jones, Swansea University, Professor Carolyn Wallace & Dr Mark Davies, University of South Wales, Professor Rachel North, Cardiff University, Mrs Rhianon Reynolds, ABUHB, Mrs Sali Davis, Optometry Wales & Dr Bablin Molik, Sight Cymru.

This project is funded by Health Care Research for Wales, Research for Patient Benefit grant.

University of South Wales Prifysgol De Cymru | SCHE Swansea Centre for Health Economics Research led, Analysis Driven, Real World Perspective | sight cymru | Canolfan PRIME Cymru PRIME Centre Wales | Cardiff University Prifysgol Caerdydd | Optometry Wales
